# Association of chronic fatigue syndrome with premature telomere attrition

**DOI:** 10.1186/s12967-018-1414-x

**Published:** 2018-02-27

**Authors:** Mangalathu S. Rajeevan, Janna Murray, Lisa Oakley, Jin-Mann S. Lin, Elizabeth R. Unger

**Affiliations:** 1 Division of High-Consequence Pathogens & Pathology, Centers for Disease Control and Prevention, 1600 Clifton Road, Atlanta, GA 30329 USA; 2 Influenza Division, Centers for Disease Control and Prevention, Atlanta, USA; 3 College of Public Health and Human Services, Oregon State University, Corvallis, USA

**Keywords:** Telomere attrition, Chronic fatigue syndrome, Myalgic encephalomyelitis, Immunosenescence, Stress

## Abstract

**Background:**

Chronic fatigue syndrome (CFS), also known as myalgic encephalomyelitis (ME), is a severely debilitating condition of unknown etiology. The symptoms and risk factors of ME/CFS share features of accelerated aging implicated in several diseases. Using telomere length as a marker, this study was performed to test the hypothesis that ME/CFS is associated with accelerated aging.

**Methods:**

Participant (n = 639) data came from the follow-up time point of the Georgia CFS surveillance study. Using the 1994 CFS Research Case Definition with questionnaire-based subscale thresholds for fatigue, function, and symptoms, participants were classified into four illness groups: CFS if all criteria were met (n = 64), CFS-X if CFS with exclusionary conditions (n = 77), ISF (insufficient symptoms/fatigue) if only some criteria were met regardless of exclusionary conditions (n = 302), and NF (non-fatigued) if no criteria and no exclusionary conditions (n = 196). Relative telomere length (T/S ratio) was measured using DNA from whole blood and real-time PCR. General linear models were used to estimate the association of illness groups or T/S ratio with demographics, biological measures and covariates with significance set at p < 0.05.

**Results:**

The mean T/S ratio differed significantly by illness group (*p* = 0.0017); the T/S ratios in CFS (0.90 ± 0.03) and ISF (0.94 ± 0.02) were each significantly lower than in NF (1.06 ± 0.04). Differences in T/S ratio by illness groups remained significant after adjustment for covariates of age, sex, body mass index, waist–hip ratio, post-exertional malaise and education attainment. Telomere length was shorter by 635, 254 and 424 base pairs in CFS, CFS-X and ISF, respectively, compared to NF. This shorter telomere length translates to roughly 10.1–20.5, 4.0–8.2 and 6.6–13.7 years of additional aging in CFS, CFS-X and ISF compared to NF respectively. Further, stratified analyses based on age and sex demonstrated that the association of ME/CFS with short telomeres is largely moderated by female subjects < 45 years old.

**Conclusions:**

This study found a significant association of ME/CFS with premature telomere attrition that is largely moderated by female subjects < 45 years old. Our results indicate that ME/CFS could be included in the list of conditions associated with accelerated aging. Further work is needed to evaluate the functional significance of accelerated aging in ME/CFS.

**Electronic supplementary material:**

The online version of this article (10.1186/s12967-018-1414-x) contains supplementary material, which is available to authorized users.

## Background

Chronic fatigue syndrome (CFS), also known as myalgic encephalomyelitis (ME) or systemic exertion intolerance disease, is a severely debilitating condition of unknown etiology [1]. Clinically, ME/CFS is characterized by chronic (longer than 6 months) multi-system symptoms including post-exertional malaise (PEM), unrefreshing sleep, significant fatigue, pain, muscle weakness, and cognitive impairment [1–3]. Proposed risk factors for ME/CFS include altered immunity, infection, environmental exposures, allergies, genetics, as well as physiological and psychological stress acting through alterations in immune and inflammatory responses [1]. The symptoms and risk factors for ME/CFS have features in common with those for accelerated aging/premature immunosenescence. Accelerated aging has been implicated in several diseases and poor health outcomes [4–10] and telomere length is a widely used marker of accelerated aging [11].

Telomeres are several thousand repeats of TTAGGG nucleotide sequences that cap the ends of linear chromosomes. Telomeres shorten during every round of cell replication because of the end-replication problem, and thus telomere length represents the replicative history of cells and dictates cells’ life span [12]. Because of the gradual erosion of telomeres with each cell division, age is the strongest predictor of an individual’s telomere length. However, several reports indicate sex differences where age-adjusted leukocyte telomere length was found to be longer in adult women than men [13, 14]. Additionally, telomere length is also impacted by other genetic, epigenetic, physiological and environmental factors. These factors contribute to variability in both the absolute telomere length and the rate of telomere shortening among individuals [11]. Chronic inflammation and oxidative stress may increase telomere attrition, potentially explaining the association of accelerated telomere shortening with disease [6, 7]. We hypothesize that ME/CFS is associated with accelerated aging and that shorter telomeres will serve as a marker of this association. We tested our hypothesis based on telomere length determined using DNA from whole blood with emphasis on a number of demographic, metabolic and allostatic load variables that are likely to impact the association between ME/CFS status and telomere length [2, 4, 7, 8, 11, 15–19]. Our results demonstrate significant telomere shortening in participants with ME/CFS and other fatigue groups in the overall study sample, with stratified analyses revealing significant premature telomere attrition in fatigued female participants < 45 years old. These findings support incorporating the model of premature and accelerated immunosenescence in future studies of ME/CFS pathophysiology.

## Methods

### Data sources and study sample

Data came from the follow-up phase of a two-wave population-based longitudinal study of CFS and fatiguing illness in Georgia, USA, conducted in 2004 and 2009 [2, 20]. The study was approved by the Institutional Review Boards of the Centers for Disease Control & Prevention, Atlanta, GA and Abt Associates, Atlanta, GA. All participants gave written informed consent for participating in the study.

The current analysis focused on data from the clinical evaluation of the follow-up phase, which included a detailed medical history, physical exam, laboratory tests, and psychiatric evaluation via the Structured Clinical Interview for DSM Disorders (SCID). Participants also completed a number of questionnaires including a demographics form, the 20-item Multidimensional Fatigue Inventory (MFI-20) [21], the 36-item Short Form Health Survey (SF-36 v2) [22, 23], and the CDC Symptom Inventory (SI) [24]. Demographic information was collected during a phone interview and further confirmed at the in-person clinical evaluation. Vital signs including heart rate (bpm), systolic and diastolic blood pressure (mmHg), height, weight, and waist and hip circumferences were collected at the clinic as part of the physical exam. The derived measures, body mass index (BMI) and waist to hip ratio (WHR) were calculated [BMI = weight/height^2^ (kg/m^2^)]; [WHR = waist/hip (cm/cm)]. The analysis included biological measures from Quest Laboratory test results such as HDL cholesterol (mg/dL), triglycerides (mg/dL), fasting glucose (mg/dL), insulin (µIU/mL), C-reactive protein (CRP, mg/dL), and albumin (g/dL).

Participants were classified as CFS if they met the 1994 international research definition applied using previously described methods [2]. Those with unexplained chronic illness with insufficient symptoms/fatigue to meet all of the criteria for CFS were grouped as insufficient symptoms/fatigue (termed as ISF). Participants meeting none of the criteria for CFS were grouped as non-fatigued (NF) controls. Additionally, participants in each group were broken into those with and without exclusionary medical/psychiatric conditions [25]. Of the 751 participants who were clinically evaluated in the GA CFS surveillance study, 71 met all the criteria for CFS without exclusionary conditions (CFS group), 78 met all the criteria for CFS but with exclusionary conditions (CFS-X group), 340 met the criteria for ISF regardless of exclusionary conditions, 212 met the criteria for NF controls without exclusionary conditions, 47 met the criteria for NF with exclusions and 3 participants were classified as “indeterminate” due to incomplete information for case definition criteria. We excluded participants with indeterminate classification, NF with medical/psychiatric exclusion, those missing data on fasting glucose and those with insufficient DNA for analysis, leaving 639 participants in 4 illness groups: 64 CFS, 77 CFS-X, 302 ISF (with and without exclusionary conditions), and 196 NF without exclusionary conditions.

### Determination of relative telomere length (T/S ratio)

Relative telomere length was measured based on a widely used real-time PCR protocol [26] with modifications as described below. The assay is based on telomere-specific primers’ ability to generate a signal proportional to the total summed length of all the telomeres in the cell. This telomere signal is normalized to the signal from primers that amplify the single copy gene, *36B4* that encodes the *acidic ribosomal phosphoprotein P0* (also known as *RPLP0*). The ratio of telomere (T) signal to the single copy gene (S) signal (expressed as T/S ratio) is proportional to the average telomere length per cell. This ratio is expressed relative to a reference DNA (K562 DNA from Promega Corporation, Madison, WI) which is assigned a T/S ratio of 1.0 as it is always compared to itself.

DNA from whole blood collected in PAXgene tubes was extracted following the manufacture’s protocol (Qiagen, Valencia, CA). DNA quality and quantity were assessed by 0.8% agarose gel electrophoresis and Nanodrop spectrophotometer (Thermo Scientific, Wilmington, DE) respectively. LightCycler 480 (Roche Life Science, Indianapolis, IN) was used for real time PCR that consisted of a 20 µL reaction containing 30 ng DNA, 1X SYBR Green I Master, and 1 µM of telomere specific or *36B4* specific primers reported previously [27]. The cycling conditions were as follows: initial denaturation at 95 °C 10 min; amplification for 35 cycles consisting of 95 °C 30 s, 58 °C 10 s, 72 °C 10 s; melting 95 °C 5 s, 70 °C 1 min, 95 °C continuous; cooling 40 °C 30 s.

The standard curve consisted of twofold dilution series from 60 to 0.94 ng, and was done in quintuplicate each for the telomere (PCR efficiency 1.91) and *36B4* (PCR efficiency 1.98) assays using K562 reference DNA. The standard curves were saved externally and imported into each PCR run at the time of analysis. The 30 ng point in the standard curve was carried in each PCR run and marked as the “standard point” to link with the external standard curve. The T/S ratio can be calculated using two methods: the ΔΔCt method using the Cp (crossing point) values or the standard curve nanogram (ng) method. T/S ratios calculated by both methods in this study were highly concordant based on the strength of linear relationship (R^2^ = 99%). We used the T/S ratios calculated by the standard curve (ng) method for the analyses in this study [28].

Prior to using the T/S ratio assay with study samples, we evaluated its reproducibility and validated it in comparison to southern blot hybridization-based terminal restriction fragment (TRF) length method using T*e*lo*TAGGG* Telomere Length Assay kit (Roche Life Science). For both reproducibility and validation tests, PAXgene DNAs from 20 volunteer samples, extracted and evaluated similar to study samples were used. T/S ratios determined on 2 different days on the same set of samples were highly reproducible based on the measure of linear relationship (R^2^ = 94%) and coefficient of variation (CV, 3.30%). TRF length expressed in base pairs (bp) and T/S ratio showed a strong linear relationship (R^2^ = 0.78%) slightly higher than that previously reported (R^2^ = 68%) [26].

Quality control of the assay was done at multiple levels including evaluation of assay reproducibility and validation. Further, each reaction was run in triplicate, with both telomere and *36B4* reactions for each sample run in the same 96-well plate to minimize variability. With each PCR run, there were 11 study samples along with a representative dilution of the standard curve point (30 ng dilution), 3 volunteer DNA samples to assess plate-to-plate variability, and a negative water control. Over the 87 runs required to complete the study, the CV for the standard curve 30 ng point (reference DNA) and the 3 control volunteer DNA samples ranged from 6.15 to 8.19%. Based on this range of CV, we used a cutoff of CV ≤ 10% within sample T/S ratio to identify samples for repeat testing. In addition, a cutoff CV of ≤ 2% was used for the Cp values within sample triplicates in either the telomere or *36B4* assays. Sixty-eight samples were repeated in order to bring the T/S ratio CV to ≤ 10%. An additional 82 samples were diluted and repeated in order to fit the Cp values within the dynamic range of the standard curve. After all quality control measures, the mean CV of all tested samples (n = 705) was 0.50 and 0.23% for the Cp values of telomere and *36B4* respectively, and 4.39% for T/S ratio (median: 4.04%; range: 0.07–9.84%).

### Statistical analyses

Data were explored to assess frequency distribution and normality. Normality test results and quantile–quantile plots showed that a transformation of T/S ratios did not significantly improve normality. As previous studies have found [29], log-transformation did make T/S ratios distribution more normal-like, we compared the results using log-transformed and untransformed data in preliminary analyses and did not find any significant difference. Therefore, the results from the analysis of the untransformed T/S ratio are presented. We used general linear models to estimate the association of CFS or T/S ratio with demographics and clinical measures. We adjusted the association of T/S ratio and CFS for covariates that showed significant association with T/S ratio in this study. We summarized associations, unadjusted and adjusted for covariates, and considered p-values below 0.05 to be statistically significant. Age- and sex-stratified analyses were also performed to investigate how these demographics moderate the association between CFS status and T/S ratio. The relationship of T/S ratio to mean TRF length (y = 4235x + 5303.8) determined from the 20 volunteer subjects was used to convert T/S ratio of a sample to mean TRF length in base pairs (bp) where x represents the T/S ratio. We estimated years of additional aging based on literature value for telomere attrition of 31–63 bp/year across 20–95 years of adulthood [5].

## Results

Table 1 provides demographic and clinical characteristics of the study sample (n = 639). The sample was mostly female (75%), white (76%), living in a rural area (50%), earning an annual income higher than $40,000 (71%), with mean age of 48 years ± standard error of mean (SEM) of 0.38 and mean BMI of 28 ± 0.21 kg/m^2^. The sample was classified into four illness groups: CFS-X (12%), CFS (10%), ISF (47%), and NF (31%). The groups did not differ in their mean age, residential areas, and illness duration but differed significantly for measures of abdominal obesity (BMI and WHR), sex, race, education, income and PEM.

**Table 1:** Participants’ characteristics by illness groups

Characteristics^a^	All (n = 639)	CFS-X (n = 77, 12%)	CFS (n = 64, 10%)	ISF (n = 302, 47%)	NF (n = 196, 31%)	p-value
Age, mean (SEM)	48.07 (0.38)	49.99 (1.11)	47.75 (1.21)	48.06 (0.58)	47.44 (0.68)	0.2725
BMI, mean (SEM)	28.13 (0.21)	28.29 (0.56)	28.86 (0.68)	28.82 (0.32)	26.77 (0.36)	0.0003
WHR (SEM)	0.85 (0.00)	0.85 (0.01)	0.85 (0.01)	0.86 (0.004)	0.83 (0.01)	0.0025
Sex						0.0009
Female	477 (74.64%)	64 (83.12%)	58 (90.63%)	222 (73.51%)	133 (67.87%)	
Race						0.0127
White	488 (76.37%)	60 (77.92%)	51 (79.69%)	219 (72.51%)	158 (80.61%)	
Black	139 (21.75%)	13 (16.88%)	10 (15.63%)	79 (26.16%)	37 (18.88%)	
Other	12 (1.87%)	4 (5.19%)	3 (4.69%)	4 (1.32%)	1 (0.51%)	
Residential area						0.1621
Urban	216 (33.80%)	26 (33.77%)	22 (34.38%)	109 (36.09%)	59 (30.10%)	
Metro	106 (16.58%)	7 (9.09%)	7 (10.94%)	49 (16.23%)	43 (21.94%)	
Rural	317 (49.61%)	44 (57.14%)	35 (54.69%)	144 (47.68%)	94 (47.96%)	
Education						< 0.0001
Less than High School	34 (5.33%)	13 (16.88%)	2 (3.13%)	14 (4.65%)	5 (2.56%)	
≥ HS ≤ 2 year College	349 (54.78%)	51 (66.23%)	40 (62.50%)	177 (58.80%)	81 (41.54%)	
4-year College	110 (17.27%)	5 (6.49%)	7 (10.94%)	47 (15.61%)	51 (26.15%)	
Graduate	144 (22.60%)	8 (10.39%)	15 (23.44%)	63 (20.93%)	58 (29.74%)	
Income						< 0.0001
≤ $20,000	78 (12.74%)	22 (31.88%)	9 (14.75%)	41 (14.04%)	6 (3.16%)	
$20,001–$40,000	102 (16.67%)	9 (13.04%)	12 (19.67%)	59 (20.21%)	22 (11.58%)	
≥ $40,001	432 (70.59%)	38 (55.07%)	40 (65.57%)	192 (65.75%)	162 (85.26%)	
Illness duration in year, mean (SEM)^b^	10.99 (0.95)	9.83 (1.10)	12.40 (1.61)	Not applicable	Not applicable	0.1796
Post-exertional malaise						
Yes	191 (29.89%)	65 (84.42%)	52 (81.25%)	70 (23.18%)	4 (2.04%)	< 0.0001

^a^Values for age, BMI, WHR and illness duration are mean (SEM). Values for all other characteristics are number of participants with percentages in parenthesis

^b^Number of subjects with information on illness duration were 86, 47 and 39 corresponding to columns All, CFS-X and CFS

Table 2 provides estimates for bivariate associations with illness groups; selected biological measures included telomere length, metabolic, and allostatic load variables. Overall T/S ratio ranged from 0.269 to 4.138 (mean: 0.98; median: 0.917). The mean telomere length differed significantly by illness group (*p* = 0.0017); the T/S ratio in CFS (0.90 ± 0.03) and ISF-all (0.94 ± 0.02) groups were each significantly lower than the NF group (1.06 ± 0.04). Triglyceride level, a measure of metabolic syndrome, was significantly different among the illness groups, but other measures included in both metabolic syndrome and allostatic load (HDL, both systolic and diastolic blood pressure and fasting glucose levels) did not differ significantly. Among the measures specific to allostatic load, heart rate, insulin and CRP showed significant differences, with mean CRP level in CFS-X and CFS groups being 93% greater than in NF group.

**Table 2:** Association of biological measures with illness groups: selected measures included telomere length, metabolic, and allostatic load variables

Variable	All (n = 639)	CFS-X (n = 77)	CFS (n = 64)	ISF (n = 302)	NF (n = 196)	p-value
T/S ratio telomere length^a^	0.98 (0.01)	0.97 (0.04)	0.90 (0.03)	0.94 (0.02)	1.06 (0.04)	0.0017
Triglycerides (mg/dL)^b^	117.13 (3.51)	144.01 (12.15)	143.53 (16.4)	114.25 (4.81)	102.43 (4.62)	0.0003
HDL (mg/dL)^b,c^	53.86 (0.65)	54.45 (2.29)	52.06 (1.92)	53.31 (0.91)	55.05 (1.16)	0.5278
Heart rate (bpm)^c^	68.02 (0.35)	69.91 (1.01)	69.05 (1.2)	68.33 (0.5)	66.47 (0.6)	0.0113
Blood pressure: systolic (mmHg)^b,c^	118.52 (0.61)	120.14 (1.99)	119.34 (1.91)	118.75 (0.92)	117.28 (0.95)	0.4935
Blood pressure: diastolic (mmHg)^b,c^	74.92 (0.39)	76.29 (1.29)	74.42 (1.32)	74.97 (0.56)	74.48 (0.66)	0.5685
Fasting glucose (mg/dL)^b,c^	94.35 (0.63)	96.68 (2.02)	93.36 (1.84)	95.5 (1.04)	91.98 (0.8)	0.0501
Insulin (µIU/mL)^c^	6.29 (0.26)	7.68 (0.92)	6.91 (0.74)	6.64 (0.39)	5.01 (0.39)	0.0062
CRP (mg/dL)^c^	3.62 (0.2)	5.1 (0.76)	4.71 (0.79)	3.73 (0.28)	2.54 (0.26)	0.0003
Albumin (g/dL)^c^	4.2 (0.01)	4.23 (0.03)	4.22 (0.03)	4.18 (0.01)	4.22 (0.02)	0.2336

Values are mean (SEM) unless otherwise indicated

^a^T/S ratio of a subject is the ratio of telomere PCR signal (T) to the single copy gene PCR signal (S). The T/S ratio is proportional to the average telomere length per cell and is expressed relative to the T/S ratio of a reference DNA

^b^Metabolic syndrome variable

^c^Allostatic load variable

Estimates for bivariate associations of T/S ratio with demographic, metabolic and allostatic load variables are given in Table 3. We observed a significant inverse association of telomere length with age (β: − 0.0051, *p* = 0.0009.), measures of abdominal obesity [BMI (β: − 0.0067, *p* = 0.0145), WHR (β: − 0.4357, *p* = 0.0149)] and PEM (β: − 0.0521, *p* = 0.0153). Telomere length was also significantly associated with education attainment (*p* = 0.0288). Telomere length was not statistically significantly related to any other demographic or biological measurements, although associations with CRP (β: − 0.0054, *p* = 0.0789) and fasting glucose (β: − 0.0017; *p* = 0.0625) were close to statistical significance.

**Table 3:** Association of demographic and biological measures with telomere length

Variable	Telomere length (T/S ratio)^a^	
	β (standard error)	p-value
Age	− 0.0051 (0.0015)	0.0009
BMI	− 0.0067 (0.0027)	0.0145
WHR	− 0.4357 (0.1784)	0.0149
Sex (female vs. male)	0.0333 (0.0339)	0.3260
Race		0.6062
White	Reference	
Black	0.0326 (0.0358)	0.3633
Other	− 0.0381 (0.1089)	0.7264
Residential area		0.9128
Urban	Reference	
Rural	− 0.0016 (0.0329)	0.9607
Metro	0.0159 (0.0442)	0.7199
Education		0.0288
Less than High School	0.1176 (0.0707)	0.0970
≥ HS ≤ 2 year College	0.1016 (0.0367)	0.0059
4-year College	0.0365 (0.0470)	0.4381
Graduate	Reference	
Income		0.1298
≤ $20,000	− 0.0925 (0.0460)	0.0445
$20,001–$40,000	− 0.0226 (0.0411)	0.5821
≥ $40,001	Reference	
Post-exertional malaise		
Yes vs. no	− 0.0521 (0.0321)	0.0153
Triglycerides (mg/dL)	− 0.0002 (0.0002)	0.3426
HDL (mg/dL)	0.0004 (0.0009)	0.6233
Heart rate (bpm)	− 0.0006 (0.0017)	0.7009
Blood pressure: systolic (mmHg)	− 0.0011 (0.0010)	0.2367
Blood pressure: diastolic (mmHg)	0.0005 (0.0015)	0.7211
Fasting glucose (mg/dL)	− 0.0017 (0.0009)	0.0625
Insulin (µIU/mL)	− 0.0009 (0.0022)	0.6856
CRP (mg/dL)	− 0.0054 (0.0030)	0.0789
Albumin (g/dL)	0.0487 (0.0621)	0.4332

^a^T/S ratio of a subject is the ratio of telomere PCR signal (T) to the single copy gene PCR signal (S). The T/S ratio is proportional to the average telomere length per cell and is expressed relative to the T/S ratio of a reference DNA

We further adjusted the association of mean telomere length with illness groups for previously defined covariates: age, BMI, WHR, PEM and education attainment. Sex was also included in the adjustment because of its significant association with CFS (Table 1). The mean telomere length differences between all fatigue groups (CFS, CFS-X, ISF) and the non-fatigued group remained significant (*p* < 0.01) before and after adjustment for covariates (Table 4). Based on the adjusted group means, telomere length was shorter by 635, 254 and 424 bp in CFS, CFS-X and ISF, respectively, compared to NF group. This shorter telomere length translates to roughly 10.1–20.5, 4.0–8.2 and 6.6–13.7 years of additional aging in CFS, CFS-X and ISF compared to NF groups, respectively, in the overall study sample (Table 4).

**Table 4:** Adjusted T/S ratio means, calculated TRF length difference and accelerated aging in fatigued groups with respect to NF in the study sample (n = 639)

CFS status	Unadjusted T/S ratio means (SEM) F = 5.13**	Adjusted T/S ratio means (SEM)^a^ F = 2.61**	Mean TRF length (bp) difference with respect to NF^b,c^	Equivalent additional years in aging with respect to NF^c^
CFS	0.90 (0.03)	0.90 (0.05)	673 (635)	10.7–21.7 (10.1–20.5)
CFS-X	0.97 (0.04)	0.99 (0.05)	360 (254)	5.7–11.6 (4.0–8.2)
ISF	0.94 (0.02)	0.95 (0.03)	500 (424)	7.9–16.1 (6.6–13.7)
NF	1.06 (0.04)	1.05 (0.04)	Reference	Reference

** *p* < 0.01

^a^Adjusted for sample characteristics including age, sex, education, BMI, WHR and PEM

^b^TRF length (bp) refers to terminal restriction fragment length in base pairs

^c^Values outside and inside the parenthesis represent those based on unadjusted and adjusted mean T/S ratios, respectively

**Fig. 1:**
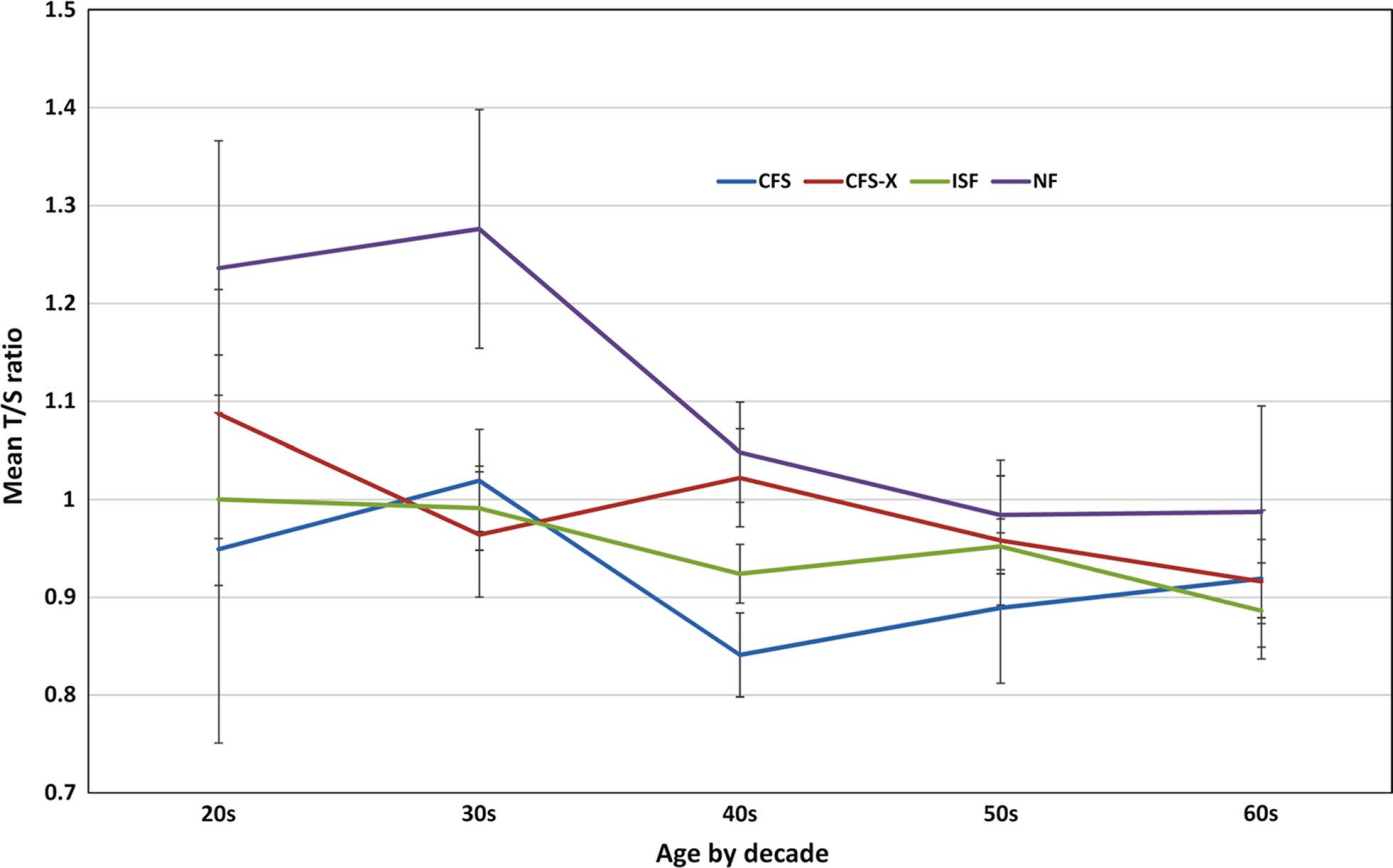
Profile of mean T/S ratio by illness group and age-by-decade in the overall study population (n = 639). Values shown are mean T/S ratio with SEM

The significant negative linear correlation between telomere length and age in the overall study sample (Table 3) was driven by the statistically significant relationship among NF participants. For participants in their second or third decade, telomere length was longest for the NF group (Fig. 1). Age trends for the NF groups indicated a shortening of 42 bp/year (β: − 0.010, *p* = 0.006) whereas the rates for the other groups were not significant [CFS (17 bp/year; β: − 0.004, *p* = 0.267), CFS-X (22 bp/year; β: − 0.005, *p* = 0.276) and ISF (8.5 bp/year; β: − 0.002, *p* = 0.226)]. As shown in Fig. 1, the significant association of telomere length with illness groups was restricted to participants < 45 years old (n = 216). For those < 45 years of age, telomeres were shorter by 932 bp in CFS, 767 bp in CFS-X and 966 bp in ISF compared to NF participants which translates to roughly 14.8–30.1, 12.2–24.7 and 15.3–31.8 years of additional aging in the CFS, CFS-X and ISF groups, respectively, compared to the NF group (Table 5).

**Table 5:** Significant telomere attrition in CFS and ISF in comparison to NF in analysis restricted to participants under 45 years old

CFS-status (participants < 45 years old, n = 216)	T/S ratio			Mean TRF length (bp) difference with respect to NF^a^	Equivalent additional years in aging with respect to NF
	β	SE	p-value		
CFS (n = 24) vs. NF (71)	− 0.220	0.0927	0.018	932	14.8–30.1
CFS-X (n = 19) vs. NF	− 0.175	0.1014	0.085	767	12.2–24.7
ISF (n = 102) vs. NF	− 0.227	0.0607	< 0.0001	966	15.3–31.2

Data not shown for participants ≥ 45 years old (n = 423) since there was no significant difference in telomere length between illness groups

^a^TRF length (bp) refers to terminal restriction fragment length in base pairs

**Fig. 2:**
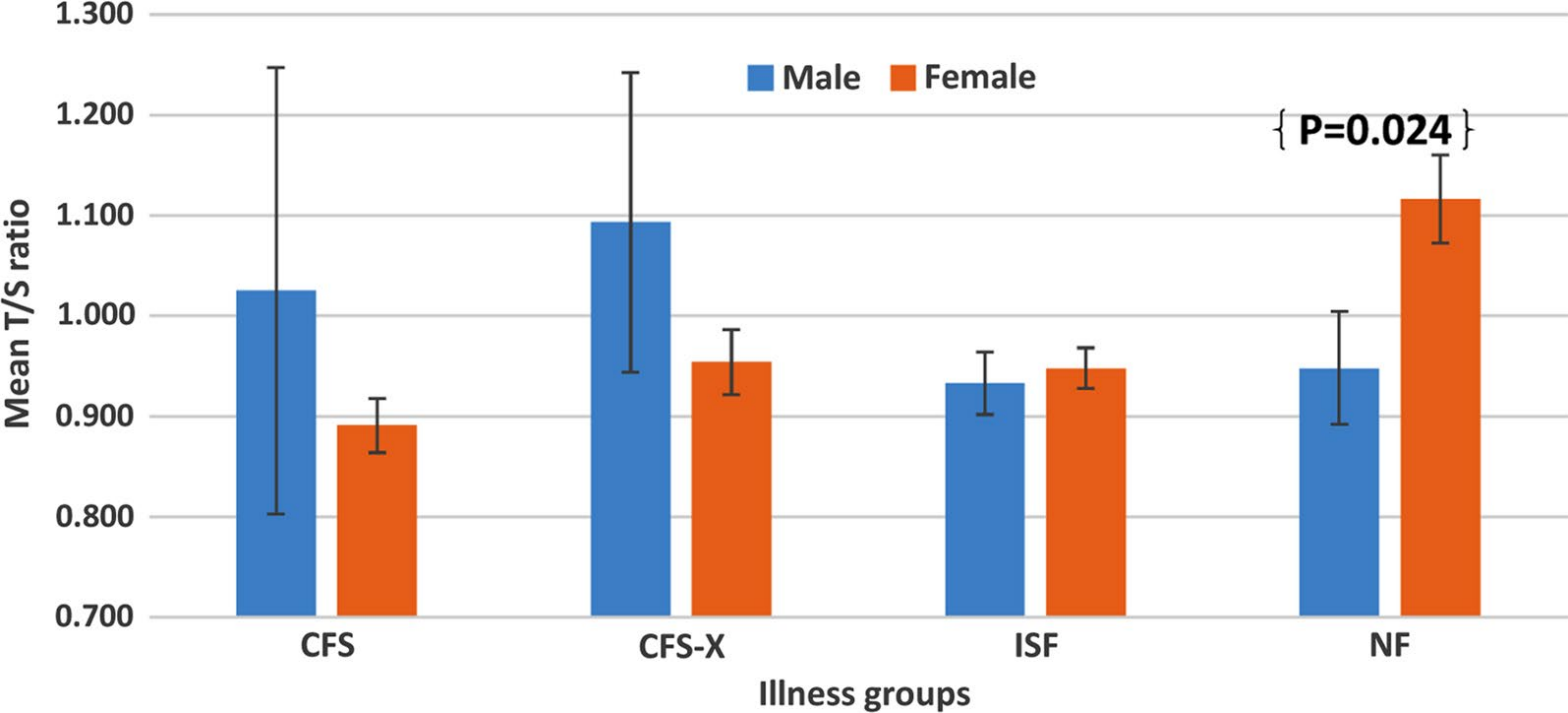
Profile of mean T/S ratio stratified by sex and by illness group in the overall study population (n = 639). Values shown are mean T/S ratio with SEM

While there was no association between telomere length and sex in the overall study sample (Table 3), the expected male/female difference was seen in the NF group; telomeres were 713 bp longer for women (n = 133; T/S ratio 1.116) than males (n = 63; T/S ratio 0.9476) (Fig. 2). Analyses stratified on sex (Table 6) showed that the significant association of telomere length with illness groups was restricted to females (n = 477). Within female participants, telomeres in the CFS group (n = 58) were shorter by 957 bp (β: − 0.226, *p* < 0.001) compared to the NF group (n = 133). Relative to NF, the difference was 690 bp in CFS-X (n = 64; β: − 0.163, *p* = 0.003) and 711 bp in ISF (n = 222; β: − 0.168, *p* < 0.001) groups, respectively. In support of this sex-specific effect, telomere length remained without significant difference among male participants in analysis that combined all males (n = 99) in the CFS, CFS-X and ISF into one group with reference to NF (n = 63).

**Table 6:** Significant telomere attrition in CFS, CFS-X and ISF in comparison to NF in analysis restricted to female participants only

CFS-status (all female, n = 477)	T/S ratio			Mean TRF length (bp) difference with respect to NF^a^	Equivalent additional years in aging with respect to NF
	β	SE	p-value		
CFS (n = 58) vs. NF (n = 133)	− 0.226	0.0566	< 0.0001	957	15.2–30.9
CFS-X (n = 64) vs. NF	− 0.163	0.0548	0.003	690	11.0–22.3
ISF (n = 222) vs. NF	− 0.168	0.0395	< 0.0001	711	11.3–23.0

Data not shown for male participants since there was no significant difference in telomere length between illness groups in male participants (n = 162)

^a^TRF length (bp) refers to terminal restriction fragment length in base pairs

**Fig. 3:**
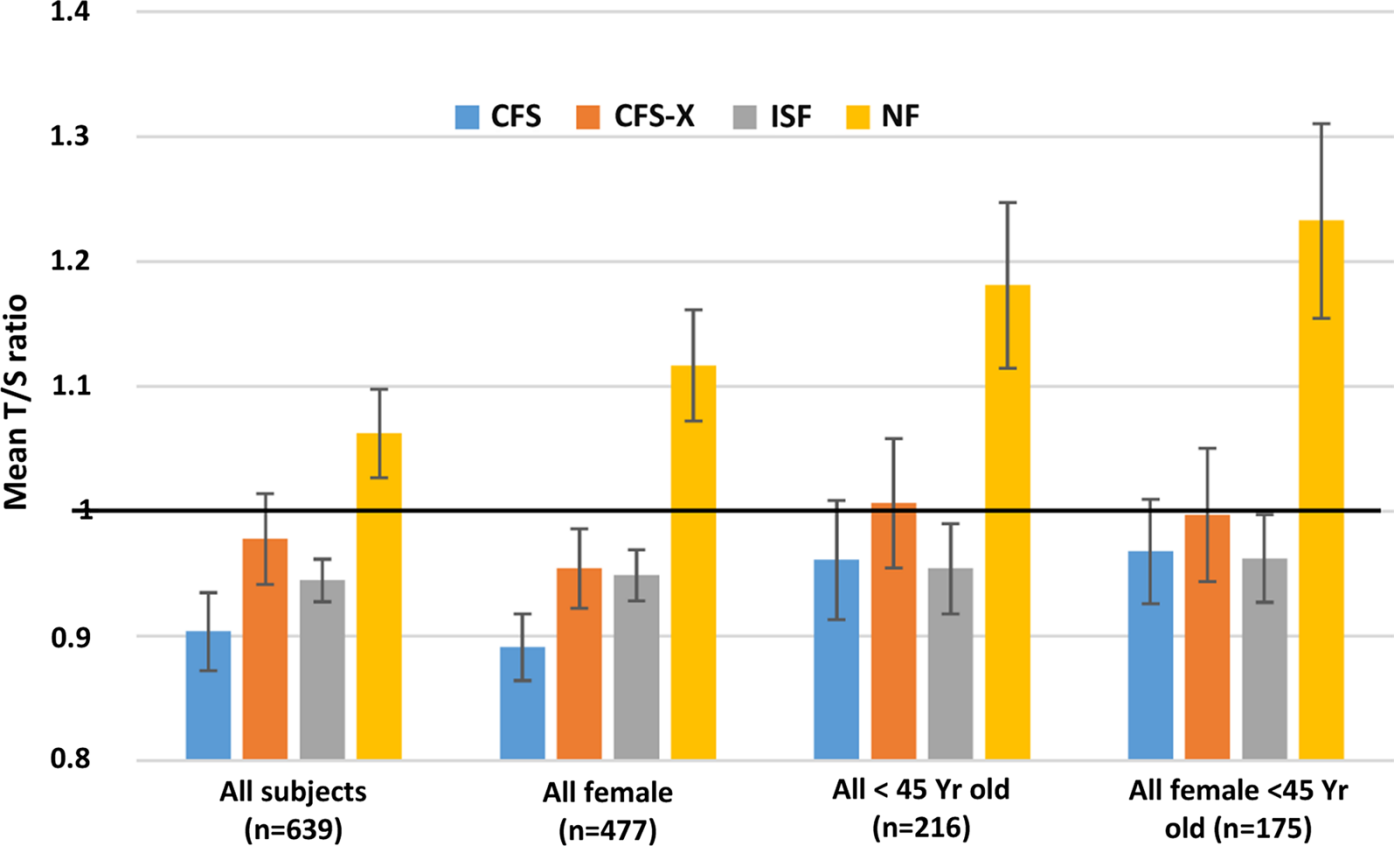
Distribution of mean T/S ratio stratified by age and sex among illness groups. Values shown are mean T/S ratio with SEM

While the mean telomere length for all illness groups was consistently lower than for the NF group in all stratified analyses, the biggest difference was seen in the subset of females < 45 years old (n = 175) (Fig. 3). The extent of telomere shortening for females < 45 years of age in each illness group compared to NF was 1130 bp in CFS, 1007 bp in CFS-X, and 1154 bp in ISF which translates to an average 17–35 years of additional aging in CFS, CFS-X and ISF. Although sample size becomes limiting with additional nested analyses, within the subset of females < 45 years old, 95% of those with CFS (21/22) had telomeres shorter than the mean telomere length (T/S ratio = 1.2324) of the NF group (OR = 15.75, 95% CI = 1.978–125.412, p = 0.0092) (Additional file 1: Figure S1).

## Discussion

To our knowledge, this is the first study demonstrating telomere attrition in patients with ME/CFS that remained significant before and after adjustment for age, sex, obesity (BMI, WHR), PEM and education attainment. Further, stratified analyses based on age and sex showed that this association of short telomeres with ME/CFS is largely moderated by female subjects < 45 years old. In agreement with studies of the general population, our study sample showed an inverse association of telomere length with age but, interestingly, the association was statistically significant only for NF participants. The age-related difference in telomere length was more pronounced before the 4th decade of life, and stratified analysis indicated that the significant association of telomere length with fatigue groups was restricted to participants < 45 years old. Similar to findings in this study, other researchers have found that premature telomere attrition was more significant in patients with lupus erythematosus [30] and rheumatoid arthritis [31] who were younger than 40–45 years old.

Overall, and in agreement with studies of the general population [32–34], telomeres were significantly longer for females compared to males in the NF group. However, this sex-related difference in telomere length was not observed among the fatigue groups (CFS, CFS-X and ISF). When the analysis of telomere length by illness classification was stratified by sex, telomere shortening in reference to NF subjects was significant only for females. However, telomere length remained without significant difference among male participants in analysis that combined all males in the CFS, CFS-X and ISF into one group with reference to NF implying that the sex-specific effect is unlikely due to difference in sample size. In the general population, the ovarian steroid hormone estrogen has been proposed to play a role in maintaining long telomeres and increased longevity in females compared to males by stimulating telomerase in specific target cells or by reducing the burden of oxidative stress due to estrogen’s effect on reactive oxygen species (ROS) [35]. Low estrogen levels were reported to be associated with short telomeres in women [36]. CFS has a higher prevalence in females and gynecologic factors such as early menopause and hysterectomy have been associated with CFS [20, 37, 38]. These prior findings along with our observation of short telomeres in female participants < 45 years old with CFS, CFS-X and ISF suggest the need for further studies on the dynamics of telomere length in relation to age, sex hormones, gynecological history and the onset of CFS.

For a wide variety of chronic diseases, elevated levels of inflammatory markers like CRP and cytokines such as interferon-γ (IFN-γ), tumor necrosis factor a (TNF-a), interleukin 1 and 6 (IL-1, IL-6), have been reported to be associated with disease progression, poor quality of life and poor therapeutic outcomes [39–45]. These reports include diabetes, chronic obstructive pulmonary disease, renal failure, psychiatric/neurological disorders, cardiovascular disease and other chronic/autoimmune/infectious diseases, including ME/CFS. Accelerated shortening of telomeres was also reported in many of these same chronic/autoimmune diseases. In most cases inflammation and telomere shortening were studied separately and a cause-effect relationship between these two processes occurring in the same cells remains to be elucidated [40, 46, 47], although it is proposed that both telomere shortening and inflammation may be involved in a bidirectional cause-effect relationship. Telomere attrition may directly act as a strong inducer of pro-inflammatory cytokines in different cell types during aging (a phenomenon called senescence associated secretory phenotypes, SASP), or that chronic inflammation may cause telomere/telomerase dysfunction directly through production of ROS resulting in telomeric DNA damage [40, 48]. Both possibilities may explain the recently reported inverse relationship of telomere length with oxidative stress and levels of IL-6 (a component of SASP) in subjects with depression [46]. It is interesting to note that senescence markers like IL-6 and p16^INK4a^ were associated with increased risk of developing cancer fatigue syndrome from cytotoxic chemotherapy [48, 49]. Elimination of senescent cells in an animal model of chemotherapy-induced fatigue almost entirely rescued normal physical activity [48, 49]. The incidence of severe fatigue in breast cancer patients correlated with the highest levels of p16^INK4a^ suggests that an increased total burden of senescent cells may cause fatigue [48, 49]. We found significant association of ME/CFS with higher levels of CRP, and marginal association of higher levels of CRP with shorter telomeres in this study. A similar inverse but stronger association of CRP and telomere length was reported recently in relation to cardiovascular disease risk [50] and obesity [51]. It is likely that pro-inflammatory cytokines may stimulate production of CRP [51, 52] that may in turn contribute to telomere shortening through oxidative stress [51, 53]. In combination with these prior studies, our observations suggest chronic inflammation and/or oxidative stress may contribute to accelerated telomere shortening or vice versa in at least a subset of subjects with ME/CFS. Further, aging-related fundamental and translational studies may provide promising avenues to assess the risk and therapeutic approach using the new class of senolytic drugs that target senescent cells to alleviate symptoms of ME/CFS [49, 54].

Mitochondrial function also declines with age, primarily in the form of impaired ATP production and increased production of ROS, both of which are associated with chronic diseases. While details of the potential inter-play between telomere shortening and mitochondrial dysfunction are unknown, many chronic diseases like metabolic, cardiovascular and neurodegenerative diseases, diabetes, mood and other psychological disorders with demonstrated telomere shortening are also associated with mitochondrial dysfunction and oxidative stress that may cause DNA damage [55]. Recent metabolomic findings [56–58] suggest ME/CFS is a hypometabolic syndrome that may relate to mitochondrial dysfunction. These studies also found the metabolomics of ME/CFS to be sex-specific [56]. Additional studies exploring the relationship between sex-specific hypometabolic state and telomere attrition could help understand the pathophysiology of ME/CFS.

There are several strengths to this explorative study on the association of telomere length with CFS. These include a relatively large overall study sample (n = 639) consisting of participants grouped into four illness status (CFS, CFS-X, ISF and NF), adjustment of illness status and telomere length with a number of demographic and biological measures, and validation of the qPCR method for reproducibility and agreement with southern blot hybridization-based TRF length method. The study also has weaknesses, including the cross-sectional design and inability to evaluate or control for duration of illness and medication used. The qPCR method provides only global mean length of telomeres and does not recognize individual short telomeres or provide information of the extend of pathologically critical short telomeres [59]. In this sense, diagnostic utility of telomere length determination by qPCR is limited but it is comparable with other methods in terms of its accuracy for association studies of telomere length with diseases [60]. A further limitation of our assay is that we used DNA extracted from whole blood consisting of lymphocytes, monocytes and granulocytes. Thus the telomere length determined in this study represents only the mean telomere length of different blood cell types instead of blood cell type-specific or telomere length distribution of different chromosomes in a given cell type.

## Conclusion

This study demonstrates a significant association of ME/CFS with premature telomere attrition, largely moderated by female subjects < 45 years old. Our results need to be replicated independently using a large sample size and with methods that can add improved diagnostic value to telomere length measurement. This observation of shorter telomeres along with reports of low- or high-grade inflammation mediated by pro-inflammatory cytokines and metabolic decline/mitochondrial dysfunction provide multiple levels of support to include ME/CFS to the list of conditions associated with accelerated aging that could be triggered by genetic, epigenetic, infection, stress or other environmental factors. Further work is needed to evaluate the functional significance, and the specific contribution of genetic, epigenetic and environmental factors to accelerated aging in ME/CFS.

## Additional file

**Additional file 1: Figure S1.** Risk for CFS in the subset of females < 45 years old based on telomere length.
