# Supplementary material for: Association of chronic fatigue syndrome with premature telomere attrition
Source: J Transl Med. 2018 Feb 27;16:44. doi: 10.1186/s12967-018-1414-x (PMC5830066; doi:10.1186/s12967-018-1414-x)

Additional File 1

Figure S1. Risk for CFS in the subset of females <45 years old (n=78) using cut-off based on the mean T/S ratio (1.2324) of the NF group in this subset (CFS, n=22 and NF, n=56). Values shown with each bar represents the number of participants with corresponding percentage of participants in the Y-axis based on the indicated cut-off.

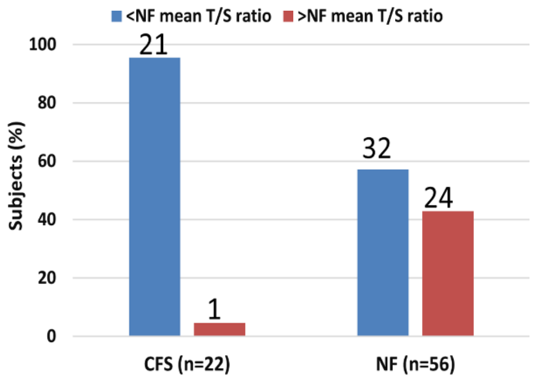

Supplement: Supplementary file 1 — Additional file 1: Figure S1. Risk for CFS in the subset of females < 45 years old based on telomere length. [file 12967_2018_1414_MOESM1_ESM.pdf]
